# Gefitinib in elderly and unfit patients affected by advanced non-small-cell lung cancer

**DOI:** 10.1038/sj.bjc.6601387

**Published:** 2003-11-11

**Authors:** C Gridelli, P Maione, V Castaldo, A Rossi

**Affiliations:** 1 Division of Medical Oncology, ‘SG Moscati’ Hospital Via Circumvallazione, Avellino 83100, Italy; 2 Direzione Sanitaria, ‘SG Moscati’ Hospital Hospital Via Circumvallazione, Avellino 83100, Italy

**Keywords:** NSCLC, gefitinib, elderly, poor PS patients

## Abstract

Elderly and poor performance status advanced non-small-cell lung cancer (NSCLC) patients often tolerate chemotherapy poorly. Special approaches are needed for these patient populations. Gefitinib (Iressa) was used in 59 elderly and/or unfit NSCLC pretreated patients participating in a compassionate use programme showing some activity and good tolerability.

More than 50% of lung cancer patients are diagnosed over the age of 65 years and about 30% over the age of 70 years. Elderly patients tolerate chemotherapy poorly compared to their younger counterpart because of the progressive reduction of organ function and comorbidities related to age (Gridelli *et al*, 2002).

We previously showed, in a randomised phase III trial (ELVIS – Elderly Lung cancer Vinorelbine Italian Study), that single-agent vinorelbine improves quality of life and survival as compared to supportive care alone in advanced NSCLC elderly patients (Elderly Lung cancer Vinorelbine Italian Study Group, 1999). More recently, in a large randomised phase III trial (MILES – Multicenter Italian Lung cancer in the Elderly Study), we did not find any advantage for combination chemotherapy with gemcitabine plus vinorelbine as compared to single-agent vinorelbine or gemcitabine (Gridelli *et al*, 2003b). Therefore, single-agent chemotherapy is considered by many as the standard treatment for advanced NSCLC elderly patients.

For patients with poor Eastern Cooperative Oncology Group performance status (ECOG PS 2), there is no treatment widely accepted as standard, and oncologists have to choose among several treatment options ranging from best supportive care to platinum-based combination chemotherapy (Bunn, 2002; Gridelli *et al*, 2003a). However, these patients usually experience severe chemotherapy-induced toxicity. Furthermore, several patients at diagnosis have major comorbidities contraindicating any chemotherapy. The possibility to develop and use well-tolerated new targeted therapies even as first-line treatment in these groups of patients is of great interest.

The epidermal growth factor receptor (EGFR) autocrine pathway contributes to a number of processes important to cancer development and progression, including cell proliferation, apoptosis, angiogenesis, and metastatic spread (Ciardiello and Tortora, 2001).

Gefitinib (ZD1839) (Iressa) is an orally available EGFR tyrosine kinase inhibitor. The major clinical development of gefitinib has been reported for single-agent therapy in recurrent NSCLC. In fact, recently, two large randomised phase II trials, named IDEAL-1 and IDEAL-2 (Iressa Dose Evaluation in Advanced Lung cancer), evaluating the activity of two different doses of gefitinib in pretreated NSCLC patients, demonstrated that Iressa, at a daily dose of 250 mg, is active and well tolerated (Kris *et al*, 2002; Fukuoka *et al*, 2003). Response rates ranged between 20% for patients pretreated with one or two chemotherapy lines (IDEAL-1 trial) and 10% for patients pretreated with two or more chemotherapy lines (IDEAL-2 trial). Although IDEAL-1 and -2 are randomised phase II trials, they confirm gefitinib 250 mg daily as an important novel treatment option for patients with pretreated advanced NSCLC and gefitinib has been very recently licensed by Japan, Australia and United States Food and Drug Administration for use in this NSCLC setting.

Considering its good safety profile, a further prospective of gefitinib use should be developed in the treatment of special patient populations, such as PS 2–3 patients, elderly and patients with major comorbidities contraindicating any chemotherapy.

In the present paper, we report tolerability and activity of gefitinib in 59 elderly and/or PS 2 or more patients, treated within a compassionate use programme.

## MATERIALS AND METHODS

Patients with the following inclusion criteria and included in the AstraZeneca's compassionate use programme were considered for retrospective analysis: histologically or cytologically confirmed advanced NSCLC; at least one prior chemotherapy regimen or radiation therapy for advanced disease or ineligible for chemotherapy or radiotherapy for any reason, age >70 years with ECOG PS>0 or younger with PS⩾2; at least one bidimensionally measurable lesion. Adequate hepatic, renal and bone marrow function were required. All patients gave written informed consent.

Objective responses were evaluated according to World Health Organisation (WHO) criteria (Miller *et al*, 1981). Cranial, thoracic, abdominal CT scans were performed at baseline and every 2 months. Toxicity was graded according to WHO criteria before each cycle of therapy (Miller *et al*, 1981).

According to the compassionate use programme, gefitinib was administered orally at a dose of 500 mg (divided in two doses) on day 1 and then as a once daily dose of 250 mg until disease progression, the appearance of unacceptable toxicity, or patient's withdrawal of consent. Patients were instructed to take the daily dose preferably in the morning. During the treatment period, no other medication with activity against NSCLC was allowed.

## RESULTS

From September 2001 to April 2003, 59 patients, 18 elderly (age >70 years) and 41 unfit (PS>2), treated in our Institution were analysed and evaluable for response and toxicity. The characteristics of patients are reported in Table 1

**Table 1:** Characteristics of patients

	**No. of patients**		
**Characteristic**	**Elderly**	**Unfit**	**All**
Total no. of patients	18	41	59
*Sex*			
Male	17	26	43
Female	1	15	16
			
*Age (years)*			
Median	73.5	60	62
Range	70–80	38–69	38–80
			
*Performance status (ECOG)*			
1	4	0	4
2	11	29	40
3	3	12	15
			
*Stage*			
IIIB	3	4	7
IV	15	37	52
			
*Histology*			
Squamous	10	17	27
Adenocarcinoma	6	21	27
Bronchioloalveolar	1	0	1
Undefined NSCLC	1	3	4
			
*Prior chemotherapy regimen*			
None	1	1	2
1	7	16	23
2 or more	10	24	34

ECOG=Eastern Cooperative Oncology Group; NSCLC=non-small-cell lung cancer.

. Median age was 62 years (range 38–80 years); PS was one in four patients, two in 40 and three in 15; male/female in 43/16 cases; stage IIIB and IV in seven and 52 patients, respectively. The histologic types were squamous cell carcinoma in 27, adenocarcinoma in 27, bronchioloalveolar in one and undefined NSCLC in four patients. The gefitinib was administered in two patients as first-line, in 23 as second-line and in 34 as third-line treatment or more. In the previous treatments, 25 (42.3%) patients received a platinum-based regimen and 15 (25.4%) both a platinum- and a docetaxel-based regimens.

Overall, three (5%) patients remained on treatment for >3 months and six (10.1%) of them for >6 months. The most common reported adverse events were grade 1 and 2 skin changes in four (6.7%) and one (1.6%) patients, respectively. Grade 1 diarrhoea in five (8.4%) patients. Grade 2 hypertransaminasaemia in one (1.6%) patient. There were two partial responses (PR) (3.4%, 95% exact binomial confidence limit: 0.4–11.7) and seven (11.8%) stable disease with an overall control of disease in 15.2% of cases. All the responses were reported in adenocarcinoma (two females) in which the EGFR expression was not available because the diagnosis was performed with a CT-guided fine-needle ago biopsy. Median progression-free survival was 7.2 weeks (range 2.1–57.1+). Median overall survival was 18.8 weeks (range 4.5–68.7+). At the cutoff date of July 30, 2003, 12 (20.3%) patients were still alive and four (6.7%) of them on gefitinib treatment. Results are summarised in Table 2

**Table 2:** Treatment activity

	**No. of patients (%)**		
	**Elderly**	**Unfit**	**All**
Complete response	0 (0)	0 (0)	0 (0)
PR	0 (0)	2 (4.9)	2 (3.4)
			
95% exact binomial confidence limit	—	0.6–16.5	0.4–11.7
Stable disease	2 (11.1)	5 (12.2)	7 (11.8)
Overall control of disease	2 (11.1)	7 (17.1)	9 (15.2)
Median progression-free survival (weeks)	7.5	7.0	7.2
			
Range (weeks)	2.4–24.8+	2.1–57.1+	2.1–57.1+
Overall survival (weeks)	19.2	18.2	18.8
			
Range (weeks)	4.5–47.5+	5.1–68.7+	4.5–68.7+

.

## DISCUSSION

Elderly and unfit patients with advanced NSCLC often are unsuitable for standard chemotherapy, because of their sensitiveness to treatment toxicity. In recent years, the rapidly expanding knowledge of cancer molecular pathogenesis has provided new targets for anticancer treatment. Some new biologic agents, such as gefitinib, have characteristics allowing their evaluation in this special patient population: low toxicity profile and oral formulation, both with potential advantages for patients.

In the present paper, we report an excellent safety profile for gefitinib in a series of 59 elderly and/or poor PS patients. No grade 3 or 4 adverse events have been registered. This toxicity profile is clearly better as compared to that reported with second- and third-line chemotherapy, even in younger and fit patients (Fossella *et al*, 2000; Shepherd *et al*, 2000). It is to underline that nearly all patients included in this analysis were heavily pretreated, thus being potentially at higher risk of treatment toxicity. Moreover, about 95% of our patients had PS⩾2, while in the IDEAL-1 trial only 12% of patients had PS 2 (Fukuoka *et al*, 2003). The reported antitumour activity (3.4% of PR and 15.2% of overall disease control) is to be considered modest, but achieved in heavily pretreated patients with no further therapeutic options.

In our opinion, single-agent gefitinib is worthy of testing in the near future for elderly and poor PS patients with advanced NSCLC even as first-line treatment. The present retrospective analysis may constitute an impulse for prospective clinical trials.

## References

[bib1] Bunn Jr PA (2002) Chemotherapy for advanced non-small-cell lung cancer: who, what, when, why? J Clin Oncol 20(18 Suppl.): 23S–33S 12235221

[bib2] Ciardiello F, Tortora G (2001) A novel approach in the treatment of cancer: tar-geting the Epidermal Growth Factor Receptor. Clin Cancer Res 7: 2958–2970 11595683

[bib3] Elderly Lung cancer Vinorelbine Italian Study Group (1999) Effects of vinorelbine on quality of life and survival of elderly patients with advanced non-small-cell lung cancer. J Natl Cancer Inst 91: 66–72 9890172 10.1093/jnci/91.1.66

[bib4] Fossella FV, DeVore R, Kerr RN, Crawford J, Natale RR, Dunphy F, Kalman L, Miller V, Lee JS, Moore M, Gandara D, Karp D, Vokes E, Kris M, Kim Y, Gamza F, Hammershaimb L (2000) Randomized phase III trial of docetaxel versus vinorelbine or ifosfamide in patients with non-small cell lung cancer previously treated with platinum-containing chemotherapy regimens. The TAX 320 Non-Small-Cell Lung Cancer Study Group. J Clin Oncol 18: 2354–2362 10856094 10.1200/JCO.2000.18.12.2354

[bib5] Fukuoka M, Yano S, Giaccone G, Tamura T, Nakagawa K, Douillard JY, Nishiwaki Y, Vansteenkiste J, Kudoh S, Rischin D, Eek R, Horai T, Noda K, Takata I, Smit E, Averbuch S, Macleod A, Feyereislova A, Dong RP, Baselga J (2003) Multi-institutional randomized phase II trial of gefitinib for previously treated patients with advanced non-small-cell lung cancer. J Clin Oncol 21: 2237–2246 12748244 10.1200/JCO.2003.10.038

[bib6] Gridelli C, Gallo C, Shepherd FA, Illiano A, Piantedosi F, Robbiati SF, Manzione L, Barbera S, Frontini L, Veltri E, Findlay B, Cigolari S, Myers R, Ianniello GP, Gebbia V, Gasparini G, Fava S, Hirsh V, Bezjak A, Seymour L, Perrone F (2003a) Gemcitabine plus vinorelbine compared with cisplatin plus vinorelbine or cisplatin plus gemcitabine for advanced non-small-cell lung cancer: a phase III trial of the Italian GEMVIN investigators and the National Cancer Institute of Canada Clinical Trials Group. J Clin Oncol 21: 3025–3034 12837810 10.1200/JCO.2003.06.099

[bib7] Gridelli C, Maione P, Colantuoni G, Rossi A (2002) Chemotherapy of non-small cell lung cancer in elderly patients. Curr Med Chem 9: 1487–1495 12171559 10.2174/0929867023369565

[bib8] Gridelli C, Perrone F, Gallo C, Cigolari S, Rossi A, Piantedosi FV, Barbera S, Ferraù F, Piazza E, Rosetti F, Clerici M, Bertetto O, Robbiati SF, Frontini L, Sacco C, Castiglione F, Favaretto A, Novello S, Migliorino MR, Gasparini G, Galetta D, Iaffaioli RV, Gebbia V (2003b) Chemotherapy for elderly patients with advanced non-small-cell lung cancer: the Multicenter Italian Lung Cancer in the Elderly Study (MILES) phase III randomized trial. J Natl Cancer Inst 95: 362–372 12618501 10.1093/jnci/95.5.362

[bib9] Kris MG, Natale BR, Herbst RS, Lynch TJ, Prager D, Belani CP, Schiller JH, Kelly K, Spiridonidis C, Albain KS, Brahmer JR, Sandler A, Crawford J, Lutzker SG, Lilenbaum R, Helms L, Wolf M, Averbuch S, Ochs J, Kay A (2002) A phase II trial of ZD 1839 (Iressa) in advanced non-small cell lung cancer (NSCLC) patients who had failed platinum- and docetaxel-based regimens (IDEAL 2). Proc Am Soc Clin Oncol 21: 292a

[bib10] Miller AB, Hoogstraten B, Staquet M, Winkler A (1981) Reporting results of cancer treatment. Cancer 47: 207–214 7459811 10.1002/1097-0142(19810101)47:1<207::aid-cncr2820470134>3.0.co;2-6

[bib11] Shepherd FA, Dancey J, Ramlau R, Mattson K, Gralla R, O'Rourke M, Levitan N, Gressot L, Vincent M, Burkes R, Coughlin S, Kim Y, Berille J (2000) Prospective randomized trial of docetaxel versus best supportive care in patients with non-small-cell lung cancer previously treated with platinum-based chemotherapy. J Clin Oncol 18: 2085–2103 10.1200/JCO.2000.18.10.2095 10811675

